# Early Antibiotic Prophylaxis in Comatose Patients to Prevent Early-Onset Ventilator-Associated Pneumonia: A Systematic Review and Bayesian Meta-Analysis

**DOI:** 10.3390/antibiotics15060622

**Published:** 2026-06-19

**Authors:** Riccardo Antolini, Filippo Violini, Roberta Domizi, Elisa Damiani, Erica Adrario, Abele Donati, Andrea Carsetti

**Affiliations:** 1 Department of Biomedical Sciences and Public Health, Università Politecnica delle Marche, via Tronto 10/A, 60126 Ancona, Italy; r.antolini@pm.univpm.it (R.A.); elisa.damiani@staff.univpm.it (E.D.); e.adrario@univpm.it (E.A.); a.donati@univpm.it (A.D.); a.carsetti@staff.univpm.it (A.C.); 2 Anesthesia and Intensive Care Unit, Azienda Ospedaliero Universitaria delle Marche, via Conca 71, 60126 Ancona, Italy; 3 Cardiac Anesthesia and Intensive Care Unit, Azienda Ospedaliero Universitaria delle Marche, 60126 Ancona, Italy

**Keywords:** ventilator-associated pneumonia, antibiotic prophylaxis, prevention, comatose

## Abstract

**Background**: Early-onset ventilator-associated pneumonia (EO-VAP) is a frequent complication in comatose patients requiring endotracheal intubation. This systematic review and Bayesian meta-analysis assesses the effectiveness of antibiotic prophylaxis in preventing EO-VAP in this population. **Methods**: Randomized controlled trials (RCTs) and observational studies enrolling adult comatose patients (GCS ≤ 8) requiring endotracheal intubation and reporting EO-VAP incidence, late-onset VAP, ICU mortality, 28-day mortality, or ICU length of stay were included. Studies without a control group or not focused on comatose patients were excluded. Following PRISMA 2020 guidelines, a comprehensive search was conducted across three databases from inception to 31 March 2026. Risk of bias was assessed using the RoB2 tool for RCTs and the ROBINS-I tool for observational studies. **Results**: In accordance with Cochrane Handbook recommendations, only RCTs were included in the quantitative analysis. Five RCTs (735 patients) demonstrated a significant reduction in EO-VAP incidence with antibiotic prophylaxis (RR 0.46 [95% CI: 0.35–0.59], *p* = 0.001, I^2^ = 0%), with the strongest effect in neurological patients (RR 0.41 [95% CI: 0.32–0.53], NNT = 5.4). No significant effect on mortality was demonstrated. Bayesian analysis confirmed these findings (posterior median RR 0.44 [95% CrI: 0.33–0.59], P(benefit) = 100%). **Limitations**: The analysis was limited by the small number of RCTs and the absence of data on antimicrobial resistance. **Conclusions**: Antibiotic prophylaxis reduces EO-VAP incidence in comatose patients, particularly neurological patients. A general recommendation cannot currently be made pending further evidence on mortality and antimicrobial resistance. **Registration:** This systematic review was prospectively registered on PROSPERO (CRD42024580280).

## 1. Introduction

Despite the progress in recent years, ventilator-associated pneumonia (VAP) remains the leading cause of healthcare-associated infections in critically ill patients [1]. VAP is defined as pneumonia occurring after 48 h of endotracheal intubation [2], caused by colonization of the upper airways followed by bacterial replication in the lower respiratory tract.

VAP can be classified into early-onset VAP (EO-VAP), which occurs within the first 4 days of intubation, and late-onset VAP (LO-VAP), which develops after this period [2]. This classification is relevant because the pathogens involved and the prognosis may differ. EO-VAP is more likely caused by antibiotic-susceptible bacteria [3]. According to different reports, it has varying effects on mortality, duration of mechanical ventilation, and length of hospital stay [4,5].

EO-VAP is common in comatose patients. In patients with traumatic brain injury, the incidence of EO-VAP ranges from approximately 20% to 40% [5,6] and accounts for about 70% of all pneumonia cases [7]. On the other hand, in the general critically ill population, 62% of VAP cases are of late onset [8].

Several risk factors are associated with an increased risk of EO-VAP [3]. Leakage of colonized subglottic secretions around the cuff, causing micro-aspiration, combined with brain injury-induced immunosuppression, may contribute to this condition [3]. In patients with reduced consciousness, EO-VAP may instead result from aspiration before intubation [3].

In this context, traditional preventive measures may fail to protect high-risk patients from pneumonia [9]. For this reason, it may be useful to evaluate the efficacy of antibiotic prophylaxis in preventing VAP.

Previous meta-analyses have shown that antibiotic prophylaxis reduces the risk of both EO-VAP and overall VAP [10,11,12]. However, no mortality reduction has been demonstrated. These findings suggest a potential benefit of prophylactic antibiotic strategies in mitigating the incidence of these significant complications in mechanically ventilated patients. This systematic review and Bayesian meta-analysis aimed to assess the effectiveness of antibiotic prophylaxis in preventing EO-VAP in comatose patients requiring endotracheal intubation, considering the new evidence available.

## 2. Methods

### 2.1. Protocol and Guidance for Conducting and Reporting

This protocol was written according to the Preferred Reporting Items for Systematic Review and Meta-Analysis Protocols (PRISMA-P) guidelines. The Preferred Reporting Items for Systematic Reviews and Meta-Analyses (PRISMA) guidelines were followed for the methodology and reporting of the systematic review (Table S1) [13].

This protocol (Supplementary Material) was registered on the International Prospective Register of Systematic Reviews (PROSPERO) (CRD42024580280).

### 2.2. Review Questions and Hypothesis

The primary review question was whether systemic antibiotics administered early after tracheal intubation prevent EO-VAP.

We hypothesized that systemic antibiotic treatment could reduce the incidence of EO-VAP.

### 2.3. Eligibility Criteria

We considered adults who were critically ill patients (≥18 years old) admitted to the ICU and intubated for a comatose state due to acute neurological impairment. For subgroup analyses, patients were classified into two categories based on the etiology of coma: (1) neurological patients, defined as primary acute brain injury, including traumatic brain injury, ischemic or hemorrhagic stroke, post-neurosurgical states, and other structural causes of coma; and (2) post-cardiac arrest patients, defined as resuscitated from out-of-hospital or in-hospital cardiac arrest, regardless of the underlying cardiac etiology. This distinction reflects the different pathophysiological mechanisms underlying aspiration and immune dysfunction in the two populations, which may influence the effectiveness of antibiotic prophylaxis. Studies on patients with pre-existing pulmonary infection at ICU admission were excluded.

Papers comparing patients receiving antibiotic prophylaxis after endotracheal intubation for EO-VAP prevention with those not receiving it were considered. Studies were excluded if they did not differentiate between EO-VAP and LO-VAP, did not focus on comatose patients, or lacked clear outcome measures.

EO-VAP, as defined by the single study, was considered.

For the secondary outcome, LO-VAP, ICU LOS, and mortality, as defined by the single study, were recorded when available.

Randomized controlled trials (RCTs) and observational studies (prospective and retrospective) were included in the systematic review without language restriction. Conference proceedings, abstracts, and case reports were excluded.

### 2.4. Search Strategy

We searched the Medline, Scopus, and Web of Science databases from their inception to April 2026. The search strategy is reported in the Supplementary Material. Reference lists of eligible studies and review articles were assessed.

### 2.5. Study Selection and Data Extraction

Two trained researchers (R.A. and F.V.) independently reviewed the titles/abstracts of all papers identified. They then independently evaluated the full text of the papers selected from this initial screening. The same researchers also independently carried out the data extraction. Any disagreements that occurred during the selection or data extraction process were resolved by mutual agreement or by involving a third independent researcher (A.C.). The screening and assessment of papers were conducted using CADIMA software version 2.2.3 (JKI—Julius Kühn-Institut).

Using a standardized data form, several data elements were extracted from the included studies, including population characteristics, type and timing of antibiotic prophylaxis administered, and information related to the intensive care stay, such as length of stay and mortality.

### 2.6. Assessment of Risk Bias in Included Studies

Two trained authors (R.A. and F.V.) independently assessed the overall quality of the studies selected. As per the Cochrane Handbook for Systematic Reviews of Interventions [14], the assessment of bias was conducted using the Cochrane Risk of Bias 2 (RoB 2) tool for randomized controlled trials, which examines five aspects of bias: randomization procedures, deviations from intended interventions, missing outcome data, outcome measurement, and selection of reported results. Each item was evaluated as low, unclear, or high risk of bias. The highest risk of bias shown for any item was used to determine the overall risk of bias for the study.

For non-randomized studies, bias was evaluated using the Risk Of Bias In Non-randomized Studies—of Interventions (ROBINS-I) tool, which evaluates bias across several domains, including participant selection, comparability of groups, and ascertainment of exposure or outcome. The highest risk of bias shown for any item was used to determine the overall risk of bias for the study.

### 2.7. Statistical Analysis

All studies reporting data on dichotomous or continuous outcomes were screened for inclusion. In accordance with the Cochrane Handbook recommendations [14], RCTs and observational studies were not pooled in the same quantitative analysis. The primary quantitative analysis was therefore conducted exclusively on RCTs. Observational studies were described narratively, and their individual effect estimates were reported separately.

Statistical analyses were performed using R (version 4.5.3) with the meta (version 8.2-1) and metafor (version 4.8-0) packages. For dichotomous outcomes, the Mantel–Haenszel random-effects model was used, and effects were estimated as risk ratios (RR) with 95% confidence intervals (CI), applying the Hartung–Knapp adjustment to the variance of the pooled estimator. For continuous outcomes, the inverse variance random-effects model was used, and results were expressed as mean differences (MD) with 95% CI. Between-study heterogeneity was assessed using the Cochran Q test and the I^2^ statistic (I^2^ > 50% considered substantial), with τ^2^ estimated by the REML method. A two-tailed *p*-value < 0.05 was set for statistical significance.

The primary analysis of ICU length of stay was restricted to RCTs that directly reported means and standard deviation, as mean estimates derived from median-based conversions are unreliable in studies with highly skewed distributions.

Pre-specified sensitivity analyses included: (1) restriction to RCTs enrolling exclusively neurological patients, defined as patients with primary acute brain injury (traumatic brain injury, stroke, or other structural causes of coma), excluding post-cardiac arrest patients; (2) leave-one-out analysis, performed by sequentially omitting each RCT and re-estimating the pooled effect; and (3) cumulative meta-analysis in chronological order of publication. Publication bias was assessed visually using funnel plots and using the trim-and-fill method. Egger’s regression test was not applied if the number of included RCTs was below the minimum threshold of 10 studies required for adequate statistical power.

A Bayesian random-effects meta-analysis was conducted using the bayesmeta package. An informative prior for the overall effect size (μ) was derived from the RCT subgroup of Righy et al. [10] [Normal(−0.844, SD = 0.256), prior RR = 0.43], the only previous meta-analysis addressing the same PICO framework. A HalfNormal(0, scale = 0.5) prior was specified for τ. Robustness to prior specification was confirmed using a weakly informative prior [Normal(−0.50, SD = 0.50)]. Results were expressed as posterior median RR with 95% credible intervals (CrI) and posterior probability of benefit P(RR < 1|data).

## 3. Results

### 3.1. Study Selection and Study Characteristics

**Figure 1:**
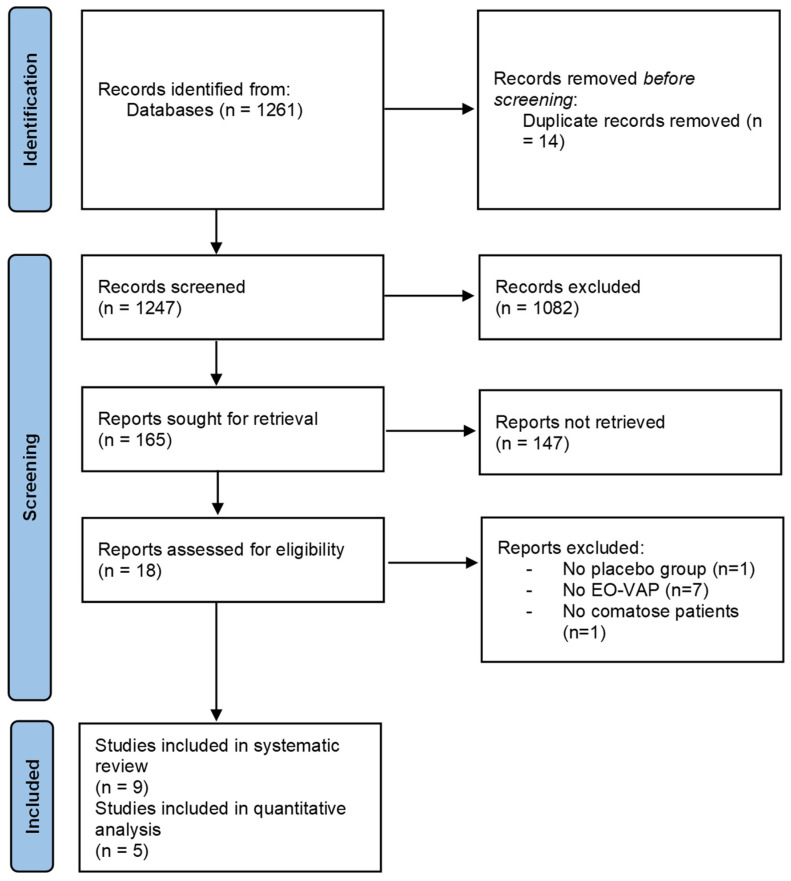
Flow diagram.

A total of 1261 titles were retrieved from the literature search. After removing duplicates (n = 14), the titles/abstracts of 1247 records were screened. A total of 1082 papers were excluded because they examined outcomes other than EO-VAP. Of the remaining 165 papers, 147 considered different populations (such as non-comatose patients) and were also excluded. After assessing 18 studies for eligibility, we were unable to retrieve nine of them because they did not include a placebo/no-antibiotic group (n = 1), did not consider EO-VAP (n = 7), or did not include comatose patients (n = 1). Nine studies were included in the qualitative analysis, considering 2056 patients. Studies were published from 1997 to 2026. The main characteristics of the selected studies are reported in Supplementary Table S2. Then, only five RCTs were included in the quantitative analysis, comprising a total of 735 patients [3,7,15,16,17] (Figure 1).

All studies included an adult population divided into two groups: a treatment group that received antibiotic prophylaxis after endotracheal intubation and a control group that did not receive antibiotic prophylaxis at the time of tracheal intubation.

### 3.2. Observational Studies

Four observational studies were included in the qualitative analysis and described narratively. Reizine et al. [18] and Claverias et al. [19] were retrospective studies, Iida et al. [20] performed a secondary analysis of a multicenter retrospective registry, and Valles et al. [21] compared a prospective cohort with a retrospective control cohort.

Reizine et al. [18] conducted a post hoc analysis of two multicenter double-blind RCTs (CORTI-TC and SPIRIT-ICU) in 295 patients with severe traumatic brain injury. Antibiotic prophylaxis, administered for surgical indications rather than VAP prevention, consisted predominantly of amoxicillin-clavulanate (72% of patients) and first- or second- generation cephalosporins (23%), with a median duration of 1 day. This prophylaxis was associated with a significant reduction in EO-VAP incidence (10% vs. 32%, RR 0.33 [95% CI: 0.19–0.56], *p* = 0.001), while late-onset VAP, ICU length of stay, and mortality did not differ between groups.

Valles et al. [21] compared a prospective cohort of patients receiving a single dose of ceftriaxone 2 g within the first 4 h of intubation with a retrospective control cohort. The incidence of EO-VAP was 2.8% in the prophylaxis group compared to 22.4% in the control group (*p* = 0.001).

Claverias et al. [19] conducted a retrospective cohort study enrolling 449 patients with a decreased level of consciousness or traumatic brain injury. Antibiotics within 24 h were administered in 53.8% of patients, predominantly amoxicillin-clavulanate (45.5%) and ceftriaxone or cefuroxime (12.8% each), for indications other than EO-VAP prophylaxis. No significant reduction in VAP incidence was observed (8.7% vs. 8.2%, *p* = 0.995). The authors interpret these results in light of the low baseline VAP incidence in their unit.

Iida et al. [20] performed a secondary analysis of the SAVE-J II multicenter retrospective registry in 448 out-of-hospital cardiac arrest patients treated with extracorporeal CPR. The antibiotic regimen used for prophylaxis was not reported in detail, which represents a significant limitation for the interpretation of the results. Prophylactic antibiotics were not significantly associated with VAP incidence (aOR 0.62 [95% CI: 0.39–1.01]), 30-day mortality, or neurological outcomes.

### 3.3. Randomized Controlled Trials

Three RCTs defined EO-VAP as ventilator-associated pneumonia occurring within 4 days of intubation [7,16,17], while the other studies defined it as occurring within 7 days of intubation [3,15].

Antibiotic regimens differed substantially across studies. Sirvent et al. [7] administered cefuroxime 1.5 g intravenously in two doses 12 h apart. Acquarolo et al. [17] used ampicillin-sulbactam 3 g every 6 h for 3 days. Mirtalaei et al. [16] administered piperacillin-tazobactam 4.5 g at the time of intubation and 12 h later. Dahyot-Fizelier et al. [3] and Valles et al. [21] both administered a single dose of ceftriaxone 2 g within 12 and 4 h of intubation, respectively. Francois et al. [15] used amoxicillin-clavulanate 1.2 g three times daily for 2 days. The duration of prophylaxis, therefore, ranged from a single dose [3,21] to three days [16], reflecting the lack of consensus on the optimal antibiotic regimen for EO-VAP prevention in this population.

### 3.4. Risk of Bias and Quality of Evidence

The quality assessment of the studies was summarized in Supplementary Material Figures S1–S4.

Among the RCTs, four studies were assessed as having some concern as an overall bias risk [3,7,15,16], while one study had an overall low risk of bias [17] (Figures S1 and S2). Regarding observational studies, three studies had a serious risk [18,19,20], and one [21] had a moderate risk of bias (Figures S3 and S4).

### 3.5. Primary Outcome

**Figure 2:**
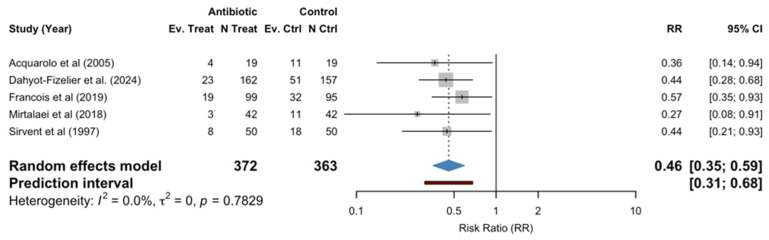
Forest plot of EO-VAP incidence in the five included RCTs [3,7,15,16,17].

Five RCTs [3,7,15,16,17] (735 patients; 180 events) reported data on EO-VAP incidence. The pooled analysis demonstrated a significant reduction in EO-VAP in the antibiotic prophylaxis group compared to controls (RR 0.46 [95% CI: 0.35–0.59], *p* = 0.001), without heterogeneity (I^2^ = 0%, τ^2^ = 0, *p* = 0.78). All five RCTs showed a consistent protective effect in favor of antibiotic prophylaxis (Figure 2).

**Figure 3:**
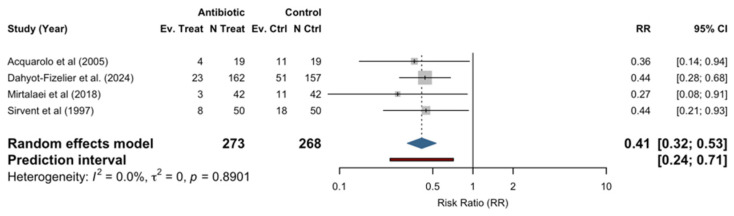
Forest plot of EO-VAP incidence restricted to RCTs enrolling exclusively neurological patients [3,7,16,17].

The four RCTs enrolling exclusively neurological patients [3,7,16,17] yielded a pooled RR of 0.41 [95% CI: 0.32–0.53], *p* = 0.0015, with complete absence of heterogeneity (I^2^ = 0%, τ^2^ = 0, Q = 0.63, *p* = 0.89) (Figure 3).

### 3.6. Secondary Outcome

#### 3.6.1. Late-Onset Ventilator-Associated Pneumonia

Three RCTs [3,15,17] (551 patients; 47 events) reported data on LO-VAP incidence. No significant reduction was observed (RR 1.18 [95% CI: 0.56–2.51], *p* = 0.440), with no heterogeneity (I^2^ = 0%, *p* = 0.60) (Figure S5).

#### 3.6.2. ICU Mortality

Four RCTs (541 patients; 123 events) reported ICU mortality data [3,7,16,17]. No significant reduction was observed in the antibiotic prophylaxis group (RR 0.75 [95% CI: 0.44–1.28], *p* = 0.182), with low heterogeneity (I^2^ = 10.3%, *p* = 0.34) (Figure S6). No RCT individually demonstrated a statistically significant reduction in ICU mortality.

#### 3.6.3. ICU Length of Stay

Three RCTs directly providing mean and standard deviation data were included in the ICU length of stay analysis [3,15,17]. The pooled mean difference was −3.93 days [95% CI: −9.02 to +1.16], *p* = 0.106, with low heterogeneity (I^2^ = 20.3%, *p* = 0.29). Statistical significance was not reached with the Hartung–Knapp correction (Figure S7).

### 3.7. Sensitivity Analyses

#### 3.7.1. Leave-One-out Analysis

The leave-one-out analysis confirmed the full robustness of the primary result. The pooled RR remained statistically significant across all five iterations, ranging from 0.41 to 0.47, with I^2^ = 0% in all scenarios and all confidence intervals excluding the null value (Figure S8).

#### 3.7.2. Recent Studies (Published from 2015 Onwards)

For the analysis restricted to RCTs published from 2015 [3,15,16], the pooled RR was 0.47 [95% CI: 0.26–0.86], I^2^ = 0%, confirming that the protective effect of antibiotic prophylaxis is present and consistent in contemporary clinical practice (Figure S9).

#### 3.7.3. Cumulative Meta-Analysis

The cumulative meta-analysis, performed in chronological order, demonstrated that statistical significance was first achieved with the addition of Mirtalaei et al. [16], when three RCTs [7,16,17] had been pooled (RR 0.38 [95% CI: 0.22–0.67], *p* = 0.018). The effect estimate remained stable with the subsequent addition of Francois et al. [15] (RR 0.47, *p* = 0.012) and Dahyot-Fizelier et al. [3] (RR 0.46 [95% CI: 0.35–0.59], I^2^ = 0%, *p* = 0.001), with I^2^ = 0% at every step of the cumulative analysis (Figure S10).

#### 3.7.4. Publication Bias

Funnel plot asymmetry was assessed visually and by using the trim-and-fill method. However, given the limited number of included RCTs (k = 5), both analyses have insufficient statistical power to reliably detect publication bias and should be interpreted with caution. The trim-and-fill method imputed two studies, yielding an adjusted pooled RR of 0.48 [95% CI: 0.38–0.61], which remained statistically significant (*p* = 0.0003) (Figures S11 and S12).

#### 3.7.5. Number Needed to Treat

Based on a baseline EO-VAP incidence of 33.9% in the control group of the included RCTs, the absolute risk reduction was 18.4% [95% CI: 13.8–21.9%], corresponding to a number needed to treat of 5.4 [95% CI: 4.6–7.3].

### 3.8. Bayesian Meta-Analysis

#### Primary Analysis—Informative Prior from Righy et al. (2017) [10]

The Bayesian random-effects meta-analysis using an informative prior derived from Righy et al. [10] [Normal(−0.844, SD = 0.256), corresponding to a prior RR of 0.43 [95% PI: 0.26–0.71]] yielded a posterior median RR of 0.44 [95% CrI: 0.33–0.59], with a posterior probability of benefit P(RR < 1|data) of 100%. The posterior heterogeneity parameter τ had a median of 0.150 (Figure S13).

The sensitivity Bayesian analysis using a weakly informative prior [Normal(−0.50, SD = 0.50), RR a priori 0.61 [95% PI: 0.23–1.62]] yielded a posterior median RR of 0.47 [95% CrI: 0.33–0.66], P(RR < 1|data) = 100%, and posterior τ median of 0.154 (Figure S14).

The convergence between the two Bayesian estimates and the frequentist results across all analyses is summarized in Table 1.

## 4. Discussion

This systematic review and meta-analysis showed that prophylactic antibiotics are effective in reducing the incidence of EO-VAP in comatose patients requiring endotracheal intubation. In accordance with Cochrane Handbook recommendations, the quantitative analysis was restricted to the five RCTs [3,7,15,16,17], yielding a pooled RR of 0.46 [95% CI: 0.35–0.59] without heterogeneity (I^2^ = 0%). The most homogeneous and clinically specific estimate was obtained from the four RCTs enrolling exclusively neurological patients (RR 0.41 [95% CI: 0.32–0.53], I^2^ = 0%), representing the primary target population for this intervention. Based on a baseline EO-VAP incidence of 33.9% in the control group of the included RCTs, this corresponds to a number needed to treat of 5.4 [95% CI: 4.6–7.3], indicating that antibiotic prophylaxis administered to approximately five comatose patients would prevent one episode of EO-VAP.

The results of the Bayesian random-effects meta-analysis support and strengthen the findings of the frequentist analysis. Using an informative prior derived from Righy et al. [10], the most pertinent previous systematic review on the same population and PICO, the posterior median RR was 0.44 [95% CrI: 0.33–0.59], with a posterior probability of benefit P(RR < 1|data) of 100%. A sensitivity Bayesian analysis using a weakly informative prior yielded a consistent estimate (RR 0.47 [95% CrI: 0.33–0.66]), confirming the robustness of the effect regardless of prior specification. The convergence between frequentist and Bayesian findings enhances the credibility of the observed effect, suggesting that the benefit of antibiotic prophylaxis is consistent across studies and generalizable across various clinical settings.

This finding is biologically plausible: in neurological patients, EO-VAP is predominantly caused by pre-intubation aspiration of colonized secretions, a mechanism directly targeted by early antibiotic prophylaxis. However, the efficacy of prophylaxis is closely linked to the oropharyngeal colonization at intubation, and in patients with pre-existing multidrug-resistant colonization, standard prophylactic regimens may provide inadequate coverage [22,23]. Notably, all included RCTs explicitly excluded patients with known MDR colonization or ongoing antibiotic therapy, limiting the generalizability of these findings to lower-risk populations. In post-cardiac arrest patients, additional pathophysiological factors—including therapeutic hypothermia, ischemia–reperfusion-induced immune dysfunction, and the use of extracorporeal membrane oxygenation—may attenuate the protective effect of prophylaxis and warrant further dedicated investigation. Of note, the two observational studies conducted in neurological patients [18,21] showed individual RRs of 0.33 and 0.13, respectively. These results are consistent with the RCT evidence.

These findings are clinically relevant given the particular vulnerability of comatose patients to EO-VAP, driven by impaired consciousness, increased aspiration risk, brain-induced immunosuppression [24,25], and sympathetic overstimulation-related capillary leakage [26]. The development of EO-VAP in this population has been associated with worse neurological functional outcomes and increased long-term mortality [27]. The current guidelines for VAP prevention recommend several interventions to reduce the incidence of VAP, including avoiding intubation, minimizing sedation, elevating the head of the bed to 30–45°, providing oral care with tooth brushing, providing early enteral nutrition, and changing the ventilator circuit only if visibly soiled or malfunctioning [18]. However, traditional VAP prevention bundles may be less effective in comatose patients, as bacterial colonization may occur early, and potentially before intubation, due to impaired airway protective reflexes. In this context, prophylactic antibiotic administration appears to protect against the progression from bacterial colonization to clinical pneumonia. However, the risk of LO-VAP was not reduced by short-course antibiotic prophylaxis (RR 1.18 [95% CI: 0.56–2.51]). Thus, LO-VAP prevention should focus on implementing the prevention bundles. A relevant source of heterogeneity across the included RCTs is the variability in EO-VAP definitions. Three RCTs defined EO-VAP as pneumonia occurring within 4 days of intubation [7,16,17], while two used a 7-day timeframe [3,15]. Studies adopting a broader definition may capture a higher absolute number of events in both groups, potentially influencing the magnitude of the effect estimate. However, the direction of the effect was consistent across all five RCTs regardless of the definition used, and the complete absence of statistical heterogeneity (I^2^ = 0%) suggests that this variability did not substantially affect the pooled estimate. This finding supports the robustness of the observed protective effect, while highlighting the need for standardized EO-VAP definitions in future trials to allow more reliable cross-study comparisons.

A further source of variability across studies was the antibiotic regimen used for prophylaxis, which ranged from a single dose of ceftriaxone [3,21] to a 3-day course of ampicillin-sulbactam [16], with other regimens including two doses of cefuroxime [7], two doses of piperacillin-tazobactam [17], and a 2-day course of amoxicillin-clavulanate [15]. Despite this heterogeneity in drug, dose, and duration, the consistency of the effect across all five RCTs and the absence of statistical heterogeneity (I^2^ = 0%) suggest that the protective effect against EO-VAP may result from early systemic antibiotic exposure rather than from any specific regimen.

The results are also supported by a recent meta-analysis by Hadley-Brown et al. [11], which demonstrated a reduction in VAP incidence with antibiotic prophylaxis in neurological patients, albeit without a corresponding reduction in mortality. The present analysis, restricted to RCTs, similarly showed no significant reduction in ICU mortality (RR 0.75 [95% CI: 0.44–1.28], *p* = 0.182), with no RCT individually demonstrating a statistically significant effect on mortality. The limited number of RCTs and events precluded adequate statistical power to detect modest differences in mortality. Similarly, ICU length of stay could not be reliably estimated from the available RCT data due to high heterogeneity (I^2^ = 87%), driven by inconsistent results across the three RCTs providing continuous data. The economic burden associated with VAP, including prolonged ICU stay and increased costs, further underscores the clinical relevance of effective prevention strategies [28].

The multicenter RCT by Dahyot-Fizelier et al. [3] was the pivotal study in this meta-analysis: the cumulative analysis demonstrated that statistical significance was first achieved with three RCTs (RR 0.38 [95% CI: 0.22–0.67], *p* = 0.018), and the effect estimate remained stable with I^2^ = 0% at every subsequent step, reaching its greatest precision after the addition of Dahyot-Fizelier et al. (RR 0.46 [95% CI: 0.35–0.59]). This stability across the cumulative analysis confirms the robustness and consistency of the evidence from RCTs.

The potential impact of antibiotic prophylaxis on antimicrobial resistance deserves careful consideration. At the individual level, none of the included RCTs reported a statistically significant increase in multidrug-resistant organisms in the prophylaxis group. The use of short-course regimens—particularly single-dose administration—has been associated with a lower risk of resistance selection compared to prolonged antibiotic exposure [29,30]. However, the limited sample sizes, short microbiological surveillance periods, and absence of systematic resistance monitoring in most studies preclude any definitive conclusion. At the ecological level, none of the included studies assessed the potential impact of prophylactic antibiotic use on local resistance patterns, as baseline data on local antimicrobial epidemiology were not reported. This is particularly relevant given that the net benefit of prophylaxis may vary substantially depending on the local prevalence of multidrug-resistant organisms—ICUs with high endemic resistance may face a fundamentally different risk-benefit balance compared to low-resistance settings [31]. In the era of antimicrobial stewardship, this represents a critical knowledge gap that must be addressed in future trials before antibiotic prophylaxis for EO-VAP prevention can be considered for routine clinical implementation.

Funnel plot asymmetry was assessed visually, given the limited number of RCTs (k = 5). The trim-and-fill method imputed two studies, yielding an adjusted pooled RR of 0.48 [95% CI: 0.38–0.61], which remained statistically significant (*p* = 0.0003). These findings suggest a possible moderate publication bias favoring studies with positive results, though the adjusted estimate remains clinically and statistically significant. It should be noted that with fewer than 10 studies, both the visual funnel plot assessment and the trim-and-fill method have limited reliability, and these analyses should therefore be interpreted with caution.

This study has several limitations. The primary quantitative analysis was restricted to five RCTs comprising 735 patients, which limits statistical power to detect modest differences in secondary outcomes such as mortality and ICU length of stay. The four observational studies included in the qualitative analysis were all judged at serious risk of bias using the ROBINS-I tool and were therefore described narratively only, in line with Cochrane Handbook recommendations against pooling RCTs and observational studies. Variability in EO-VAP definitions, diagnostic criteria, patient populations, and antibiotic regimens across studies represents a further source of heterogeneity that could not be fully addressed. The meta-analysis did not examine pathogen-specific outcomes or the optimal type, dose, and timing of antibiotic prophylaxis, which represent important areas for future research. Furthermore, none of the included studies systematically evaluated the impact of antibiotic prophylaxis on the development of multidrug-resistant organisms, nor did they report data on local antimicrobial resistance patterns at the time of the study, making it impossible to assess whether the ecological impact of prophylaxis may differ across settings with varying baseline resistance profiles. Given the growing concern about antimicrobial resistance in the ICU setting, this represents a critical knowledge gap that prevents a definitive assessment of the risk-benefit profile of this intervention and should be addressed in future research.

## 5. Conclusions

This systematic review and meta-analysis shows that antibiotic prophylaxis at intubation may probably reduce EO-VAP incidence in comatose patients, with the most robust evidence coming from RCTs in neurological patients (RR 0.41 [95% CI: 0.32–0.53], I^2^ = 0%). These results are confirmed by both frequentist and Bayesian analyses. No significant effect on mortality was demonstrated in the RCT analysis, and ICU length of stay could not be reliably estimated due to high heterogeneity across the available data.

Despite evidence that antibiotic prophylaxis reduces the incidence of EO-VAP, a general recommendation in favor of this intervention cannot currently be made, given both the lack of systematic data on its impact on antimicrobial resistance and its neutral effect on mortality. However, the consistent and robust effect observed across RCTs in neurological patients, confirmed by both frequentist and Bayesian analyses, supports the need for further large, randomized trials in this specific high-risk population to confirm our results and define the optimal antibiotic regimen.

## Figures and Tables

**Table 1:** Convergence of frequentist and Bayesian estimates of EO-VAP risk reduction with antibiotic prophylaxis.

Analysis	RR	95% CI/CrI	I^2^/τ
Frequentist—RCTs all (k = 5)	0.46	0.35–0.59	I^2^ = 0%
Frequentist—RCT neurological (k = 4)	0.41	0.32–0.53	I^2^ = 0%
Bayesian—Righy prior	0.44	0.33–0.59	τ = 0.150
Bayesian—weak prior	0.47	0.33–0.66	τ = 0.154

## Data Availability

The datasets analyzed during the current study are available from the corresponding author on reasonable request.

## Supplementary Materials

The following supporting information can be downloaded at: https://www.mdpi.com/article/10.3390/antibiotics15060622/s1, Search Strategy; Figure S1: Traffic light plot summarising the risk of bias assessment of the five included randomized controlled trials using the Cochrane Risk of Bias 2 (RoB2) tool.; Figure S2. Summary bar chart of the risk of bias assessment of the five included randomized controlled trials across all RoB2 domains, showing the proportion of studies rated as low risk, some concerns, or high risk for each domain; Figure S3. Traffic light plot summarising the risk of bias assessment of the four included observational studies using the ROBINS-I tool; Figure S4. Summary bar chart of the risk of bias assessment of the four included observational studies across all ROBINS-I domains, showing the proportion of studies rated at each risk level; Figure S5. Forest plot of LO-VAP incidence. Figure S6. Forest plot of ICU mortality. Figure S7. Forest plot of ICU LOS. Figure S8. Leave-one-out sensitivity analysis of EO-VAP incidence. Figure S9. Forest plot of EO-VAP incidence restricted to the three RCTs published from 2015 onward. Figure S10. Forest Plot of cumulative meta-analysis of EO-VAP incidence in chronological order of publication. Figure S11. Funnel plot of the five RCTs included in the primary meta-analysis. Figure S12. Funnel plot after application of the trim-and-fill method (L-estimator). Figure S13. Posterior density distribution of the effect size (log RR scale) from the Bayesian analysis using the Righy et al. prior. Figure S14. Posterior density distribution of the effect size (log RR scale) from the Bayesian sensitivity analysis; Table S1: PRISMA checklist; Table S2: Studies characteristics.
