# Supplementary material for: Early Antibiotic Prophylaxis in Comatose Patients to Prevent Early-Onset Ventilator-Associated Pneumonia: A Systematic Review and Bayesian Meta-Analysis
Source: Antibiotics (Basel). 2026 Jun 19;15(6):622. doi: 10.3390/antibiotics15060622 (PMC13295702; doi:10.3390/antibiotics15060622)
Supplement: Supplementary file 1 [file antibiotics-15-00622-s001.zip › antibiotics-4371235-supplementary.pdf]

SUPPLEMENTARY MATERIAL

Early antibiotic prophylaxis in comatose patients to prevent early-onset ventilator associate pneumonia: a systematic review and Bayesian meta-analysis

Riccardo Antolini MD,[1-2] Filippo Violini MD,[1,3] Roberta Domizi MD,PhD,[1,2*] Elisa Damiani MD,PhD,[1,2] Erica Adrario MD,[1,2] Abele Donati MD,PhD,[1,2] Andrea Carsetti MD[1,2]

[1]Department of Biomedical Sciences and Public Health, Università Politecnica delle Marche, via Tronto 10/A, 60126 – Ancona, Italy.

[2]Anesthesia and Intensive Care Unit, Azienda Ospedaliero Universitaria delle Marche, via Conca 71, 60126 – Ancona, Italy.

[3] Cardiac Anesthesia and Intensive Care Unit, Azienda Ospedaliero Universitaria delle Marche,, Ancona, Italy.

SEARCH STRATEGY

1- [“Aspiration pneumoniae” OR “Ventilator penumoniae” OR “Pneumoniae ventilator associated” OR “Ventilator associated pneumoniae” OR “VAP” OR “Respiratory infection” OR “pneumoniae”] AND [“coma” OR “Altered level of conciusness” OR “Consciousness disorder” OR “Acute brain injury” OR “Traumatic Brain injury” OR “TBI” OR “cardiac Arrest”] AND [“antibiotic prophylaxis” OR “antibiotic premedication”]
2- [“Early ventilator associated pneumoniae” OR “early-VAP” OR “EO-VAP”] AND antibiotic
3- ["ventilator associated pneumoniae" OR "VAP" OR "VAP" OR "VAC" OR "IVAC"] AND ("antibiotic" OR "antibiotic prophylaxis")

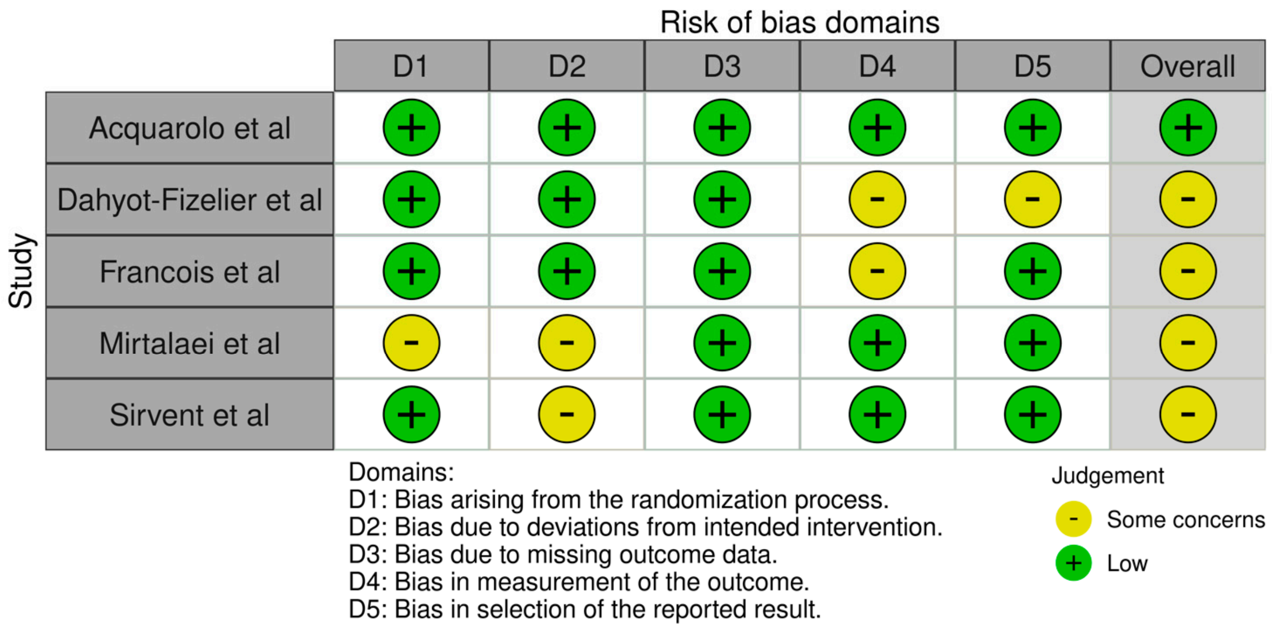

Fig. S1. Traffic light plot summarising the risk of bias assessment of the five included randomized controlled trials using the Cochrane Risk of Bias 2 (RoB2) tool.

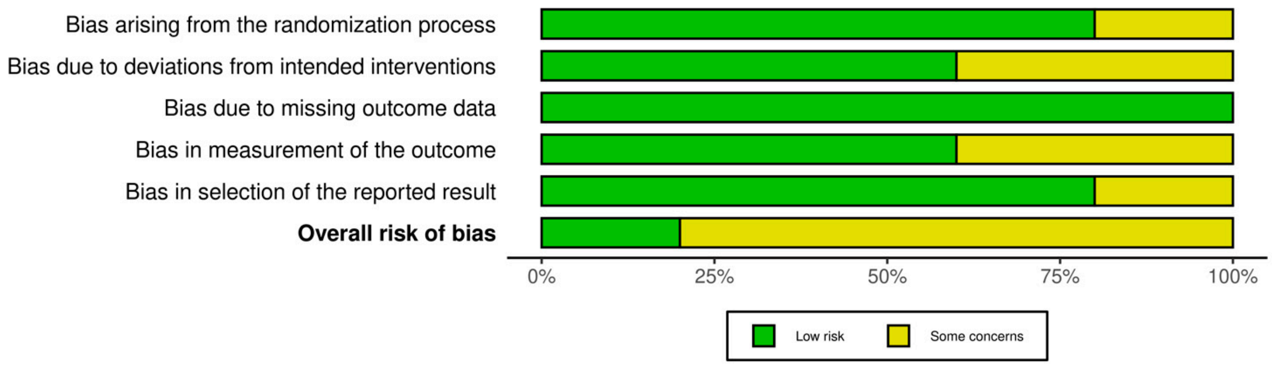

Fig. S2. Summary bar chart of the risk of bias assessment of the five included randomized controlled trials across all RoB2 domains, showing the proportion of studies rated as low risk, some concerns, or high risk for each domain.

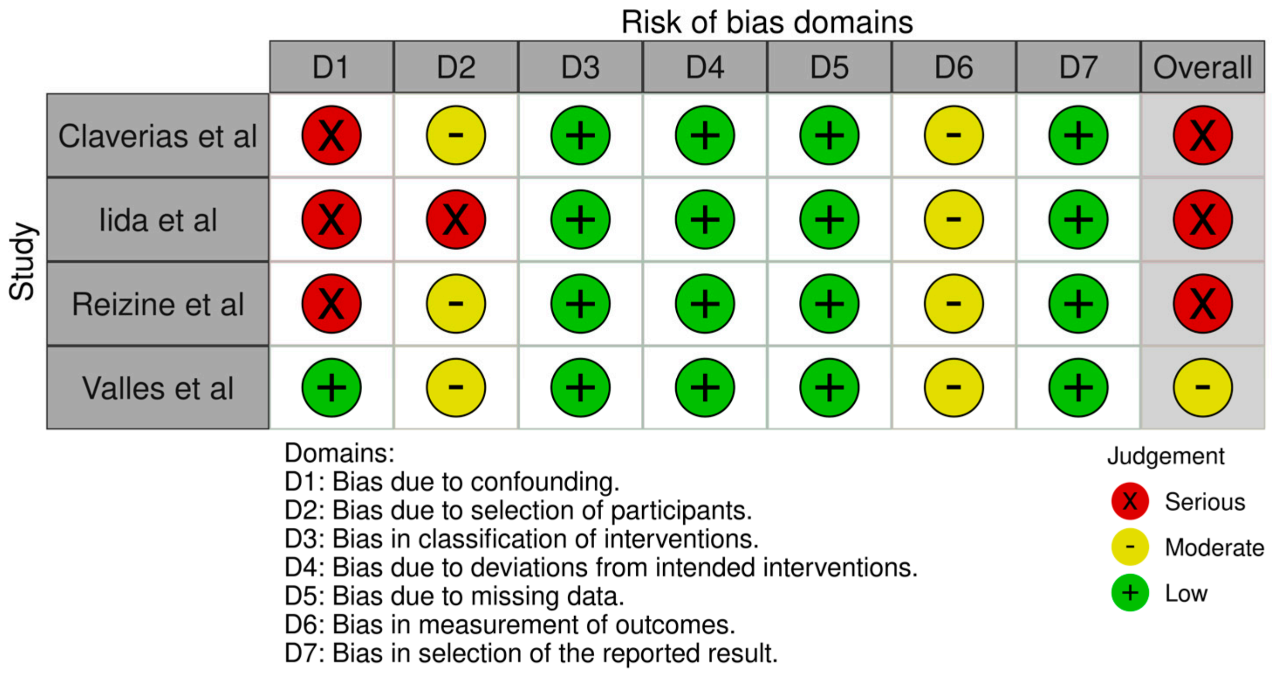

Fig. S3. Traffic light plot summarising the risk of bias assessment of the four included observational studies using the ROBINS-I tool.

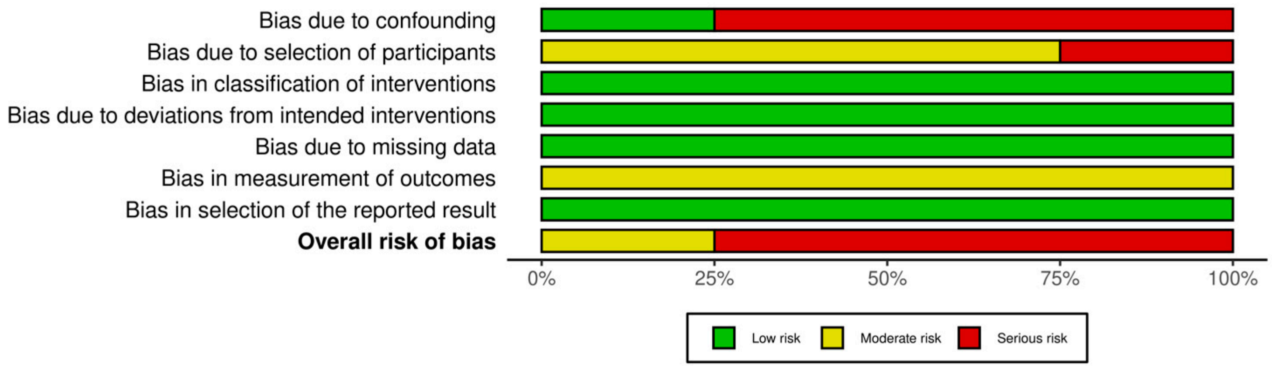

Fig S4. Summary bar chart of the risk of bias assessment of the four included observational studies across all ROBINS-I domains, showing the proportion of studies rated at each risk level.

Table S1. PRISMA checklist.

Section and Topic	Item #	Checklist item	Location where item is reported
**TITLE**			
Title	1	Identify the report as a systematic review.	1
**ABSTRACT**			
Abstract	2	See the PRISMA 2020 for Abstracts checklist.	1
**INTRODUCTION**			
Rationale	3	Describe the rationale for the review in the context of existing knowledge.	2
Objectives	4	Provide an explicit statement of the objective(s) or question(s) the review addresses.	2
**METHODS**			
Eligibility criteria	5	Specify the inclusion and exclusion criteria for the review and how studies were grouped for the syntheses.	3
Information sources	6	Specify all databases, registers, websites, organisations, reference lists and other sources searched or consulted to identify studies. Specify the date when each source was last searched or consulted.	3
Search strategy	7	Present the full search strategies for all databases, registers and websites, including any filters and limits used.	3
Selection process	8	Specify the methods used to decide whether a study met the inclusion criteria of the review, including how many reviewers screened each record and each report retrieved, whether they worked independently, and if applicable, details of automation tools used in the process.	3
Data collection process	9	Specify the methods used to collect data from reports, including how many reviewers collected data from each report, whether they worked independently, any processes for obtaining or confirming data from study investigators, and if applicable, details of automation tools used in the process.	3-4
Data items	10a	List and define all outcomes for which data were sought. Specify whether all results that were compatible with each outcome domain in each study were sought (e.g. for all measures, time points, analyses), and if not, the methods used to decide which results to collect.	4
	10b	List and define all other variables for which data were sought (e.g. participant and intervention characteristics, funding sources). Describe any assumptions made about any missing or unclear information.	4
Study risk of bias assessment	11	Specify the methods used to assess risk of bias in the included studies, including details of the tool(s) used, how many reviewers assessed each study and whether they worked independently, and if applicable, details of automation tools used in the process.	3-4
Effect measures	12	Specify for each outcome the effect measure(s) (e.g. risk ratio, mean difference) used in the synthesis or presentation of results.	4
Synthesis methods	13a	Describe the processes used to decide which studies were eligible for each synthesis (e.g. tabulating the study intervention characteristics and comparing against the planned groups for each synthesis (item #5)).	4-5
	13b	Describe any methods required to prepare the data for presentation or synthesis, such as handling of missing summary statistics, or data conversions.	3-4
	13c	Describe any methods used to tabulate or visually display results of individual studies and syntheses.	3-4
	13d	Describe any methods used to synthesize results and provide a rationale for the choice(s). If meta-analysis was performed, describe the model(s), method(s) to identify the presence and extent of statistical heterogeneity, and software package(s) used.	4
	13e	Describe any methods used to explore possible causes of heterogeneity among study results (e.g. subgroup analysis, meta-regression).	4

Section and Topic	Item #	Checklist item	Location where item is reported
	13f	Describe any sensitivity analyses conducted to assess robustness of the synthesized results.	4
Reporting bias assessment	14	Describe any methods used to assess risk of bias due to missing results in a synthesis (arising from reporting biases).	3-4
Certainty assessment	15	Describe any methods used to assess certainty (or confidence) in the body of evidence for an outcome.	3-4
**RESULTS**			
Study selection	16a	Describe the results of the search and selection process, from the number of records identified in the search to the number of studies included in the review, ideally using a flow diagram.	4
	16b	Cite studies that might appear to meet the inclusion criteria, but which were excluded, and explain why they were excluded.	4
Study characteristics	17	Cite each included study and present its characteristics.	5-6
Risk of bias in studies	18	Present assessments of risk of bias for each included study.	6
Results of individual studies	19	For all outcomes, present, for each study: (a) summary statistics for each group (where appropriate) and (b) an effect estimate and its precision (e.g. confidence/credible interval), ideally using structured tables or plots.	6-7-8
Results of syntheses	20a	For each synthesis, briefly summarise the characteristics and risk of bias among contributing studies.	6-7-8
	20b	Present results of all statistical syntheses conducted. If meta-analysis was done, present for each the summary estimate and its precision (e.g. confidence/credible interval) and measures of statistical heterogeneity. If comparing groups, describe the direction of the effect.	6-7-8
	20c	Present results of all investigations of possible causes of heterogeneity among study results.	6-7-8
	20d	Present results of all sensitivity analyses conducted to assess the robustness of the synthesized results.	6-7-8
Reporting biases	21	Present assessments of risk of bias due to missing results (arising from reporting biases) for each synthesis assessed.	6-7-8
Certainty of evidence	22	Present assessments of certainty (or confidence) in the body of evidence for each outcome assessed.	6-7-8
**DISCUSSION**			
Discussion	23a	Provide a general interpretation of the results in the context of other evidence.	8-9
	23b	Discuss any limitations of the evidence included in the review.	10
	23c	Discuss any limitations of the review processes used.	10
	23d	Discuss implications of the results for practice, policy, and future research.	9-10
**OTHER INFORMATION**			
Registration and protocol	24a	Provide registration information for the review, including register name and registration number, or state that the review was not registered.	2
	24b	Indicate where the review protocol can be accessed, or state that a protocol was not prepared.	2
	24c	Describe and explain any amendments to information provided at registration or in the protocol.	2
Support	25	Describe sources of financial or non-financial support for the review, and the role of the funders or sponsors in the review.	10

Section and Topic	Item #	Checklist item	Location where item is reported
Competing interests	26	Declare any competing interests of review authors.	10
Availability of data, code and other materials	27	Report which of the following are publicly available and where they can be found: template data collection forms; data extracted from included studies; data used for all analyses; analytic code; any other materials used in the review.	10

From: Page MJ, McKenzie JE, Bossuyt PM, Boutron I, Hoffmann TC, Mulrow CD, et al. The PRISMA 2020 statement: an updated guideline for reporting systematic reviews. BMJ 2021;372:n71. doi: 10.1136/bmj.n71. This work is licensed under CC BY 4.0. To view a copy of this license, visit https://creativecommons.org/licenses/by/4.0/

Table S2. Studies characteristics.

Study	Design	Sample size	Population	Intervention	Comparison	VAP definition	Early VAP definition	VAP Prevention Bundle
Sirvent et al. 1997 (7)	RCT	105	Head injury or coma caused by medical stroke, GCS ≤12. Subjects undergoing surgery for space occupying head lesions	Cefuroxime 1.5 g bid	No prophylaxis (antibiotics allow for other reasons)	New infiltrates plus two of the following were present: fever ≥ 38°C, leukocytosis ≥ 11,000/mm^3, purulent tracheal secretions, Pao2/Fio2 < 240 mm Hg, or clinical pulmonary infection score > 6. Confirmed by isolating a pathogen from bronchoalveolar lavage with ≥ 10^4 CFU/ml.	<4 days of MV	Stress ulcer prophylaxis (ranitidine IV + sucralfate); enteral nutrition. SDD not used. Semi-recumbent positioning and cuff pressure monitoring not specified.
Acquarolo et al. 2005 (17)	Randomized, open study	38	Adult (≥18 years) comatose (GCS ≤8) mechanically ventilated patients	Ampicillin Sulbactam (3 g every 6 h for 3 days)	Standard treatment	new and persistent chest radiographic infiltrate was associated with one of the following criteria: (1) purulent tracheo-bronchial secretions; (2) fever ≥38.3°C or hypothermia; (3) leukocytosis or leucopoenia (>10,000/mm^3 and, respectively, <5,000/mm^3). Confirmed by the	<4 days of MV	Semi-recumbent positioning (when feasible); stress ulcer prophylaxis (ranitidine IV 50mg every 6h); enteral nutrition when possible. SDD not used. Cuff pressure

						isolation of a potentially pathogenic micro-organism from bronchoscopic BAL (>10^4 cfu/ml) or non bronchoscopic protected mini-BAL (>10^4 cfu/ml).		monitoring not specified.
Valles et al. 2013 (21)	Comparative cohort study	129	Comatose patients on mechanical ventilation (Glasgow coma score ≤8)	Ceftriaxone 2 g single dose	No antibiotic prophylaxis	New or progressive infiltrates or consolidation on chest radiographs in the presence of two of the following: leukocytes 11,000/mm^3 or 4,000/mm^3, fever ≥38°C or hypothermia, <36°C, or new onset of purulent endotracheal secretions or change in character of sputum. Pneumonia was considered definite in the presence of either a quantitative culture of tracheal aspirate cfu/mL or a quantitative culture of a protected specimen brush 10^3 cfu/mL or a quantitative culture of BAL 10^4 cfu/mL.	<4 days of MV	Cuff pressure monitoring; continuous subglottic suctioning; semi-recumbent positioning; oral care with chlorhexidine. Applied uniformly in both groups throughout the study period.

Mirtalaei et al. 2019 (16)	RCT	84	Patients with stroke, >20 yo, GCS ≤8	Piperacillin-tazobactam 4.5 g at the time of intubation and 12h later	Placebo	New or progressive consolidation or infiltrates appeared on chest radiographs in the presence of two of the following: fever ≥ 38°C or hypothermia < 36°C, leukocytes ≥ 11,000/mm^3 or ≤ 4,000/mm, or new onset of purulent endotracheal secretions or change in the character of sputum. Pneumonia was considered definite in the presence of either a quantitative culture of tracheal aspirate ≥10^6 CFU/mL or a quantitative culture of a protected specimen brush ≥10^3 CFU/mL or a quantitative culture of BAL ≥ 10^4 CFU/mL	<4 days of MV	Not reported.
Francois et al. 2019 (15)	Multicenter RCT	194	Adult patients (>18 yo) hospitalized in the ICU after an out-of-hospital cardiac arrest with shockable	Amoxicillin-clavulanate 1.2 g tid for 2 days	Placebo	Criteria from 2010 Food and Drug Administration guidance for diagnosis and confirmation of ventilator associatedpneumonia	<7 days of MV	Head-of-bed elevation; daily sedation vacations and readiness-to-extubate assessment; daily oral care. Targeted

			rhythm and treated with 32-to-34°C targeted temperature management					temperature management 32–34°C in all patients.
Reizine et al. 2019 (18)	Retrospective	295	S PIRIT-ICU age >18 years, severe traumatic brain injury, expected mechanical ventilation ≥48 hours<br>CORTI TC: severe traumatic brain injury, in the first 24 hours following trauma, age between 15 and 65 years and informed consent from a next-of-kin.	SPIRIT-ICU: >12h after tracheal intubation, tetraplegia, facial trauma, pulmonary contusion involving >1 lobe, aspiration pneumonia at admission, current curative antimicrobial treatment at admission, known allergy to povidone-iodine, pregnancy.<br>CORTI-TC: treatment with corticosteroids in the previous 6 months, immunosuppression, tetraplegia, antibiotic treatment at the time of inclusion		The diagnosis of VAP had to occur at least 48 h after tracheal intubation and mechanical ventilation. Two or more of the following criteria were required: body temperature N 38 °C, purulent pulmonary secretions, leucocytosis N12,000/mL or leucopaenia b4000/mL (the level of leucocytosis and leucopaenia was N10,000/mL and b 4000/mL, respectively, in the SPIRIT-ICU), and a new or persistent pulmonary infiltrate on chest radiography. In the SPIRIT-ICU trial, an independent diagnosis validation committee blindly	>2 and <4 days of MV	Semi-recumbent positioning; cuff pressure monitoring. Applied in both groups.

						classified each patient as positive or negative for VAP, whereas this procedure was not used in the CORTI-TC study. All VAP were microbiologically documented by quantitative culture from specimen brush (≥ 103 cfu/mL), bronchoalveolar lavage (≥ 10.4 cfu/mL) or endotracheal aspirate (≥ 10.6 cfu/mL)		
Dahyot-Fizelier et al. 2024 (3)	Multicenter RCT	319	Comatose (GCS ≤12) adult (>18 yo) patients, who were predicted to require mechanical ventilation for more than 48 h after head trauma, stroke, or subarachnoid haemorrhage	Ceftriaxone 2 g single dose	Placebo	New or modification of a previously existing condensation in the presence of two of the following: fever ≥38°C or hypothermia <36°C, leukocytes ≥11,000/mm^3 or ≤4,000/mm, or new onset of purulent endotracheal secretions. A positive bacterial analysis of the respiratory tract with cultures of at least 10^3 cfu/mL blind for a brush by fibroscopy or blind	<7 days of MV	Standardised bundle: hand hygiene; head-of-bed elevation 30° (monitored every 4h); cuff pressure monitoring every 8h (25–30 cmH_2O); oral care every 8h; tracheal aspiration with sterile equipment when

						protected distal sampling, 10^4 cfu/mL for BAL, and 10^6 cfu/mL for endotracheal aspirate		required; early enteral nutrition. No SOD or SDD.
Claverias et al. 2025 (19)	Retrospective, observational, single center	449	Low level of consciousness or TBI with or without other trauma associated, who required mechanical ventilation > 48h	Administered only when indicated for other reasons	No antibiotics received	(1) Fever without other apparent cause (2) Changes inspùtum production (3) Positive respiratory culture (tracheal aspirate >1000000 CFU/mL or bronchoalveolar lavage ≥10000 CFU/mL) and (4) radiological evidence of pneumonia	Between 48h and 7 days after intubation	Mandatory measures of the SEMICYUC "Neumonía Zero" project applied throughout the study period. Specific compliance rates not available due to retrospective design.
Iida et al. 2026 (20)	Secondary analysis of the SAVE-J II study (multicenter, retrospective registry study)	919	Adult patients (at least 18 years of age) with OHCA treated with ECPR	Any antibiotic within 24h after admission (regimen not reported)	No antibiotics received	Clinical criteria (at least one of three clinical features (i.e. fever38.0°C, leukocytosis [>12,000cells/lL] and purulent tracheobronchial secretions) + radiologic criteria (new or progressive and persistent infiltrate char-	Clinical + radiological + microbiologc criteria 48-72h after hospitalization	Not reported.

						acteristic of bacterial pneumonia or the presence of a new consolidation on chest X-ray imaging) + microbiologic criteria (positive respiratory culture that did not contain normal bacterial flora)		

BAL: bronchoalveolar lavage; CFU: colony-forming units; GCS: Glascow Coma Scale; MV: mechanical ventilation; RCT: randomized controlled trial; SOD: Selective Oropharyngeal Decontamination; SDD: Selective Digestive Decontamination.

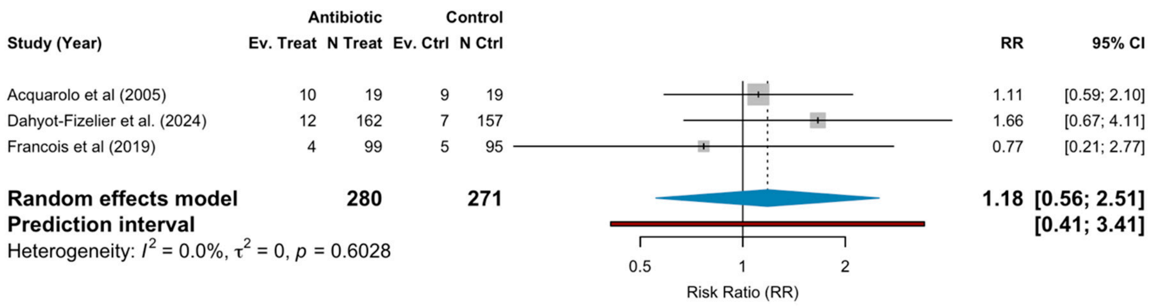

Fig. S5. Forest plot of LO-VAP incidence [3,15,17].

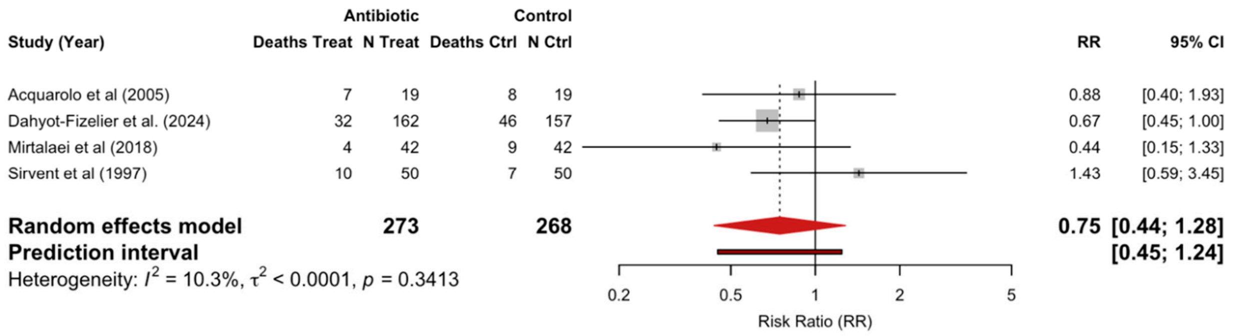

Fig. S6. Forest plot of ICU mortality [3,7,16,17].

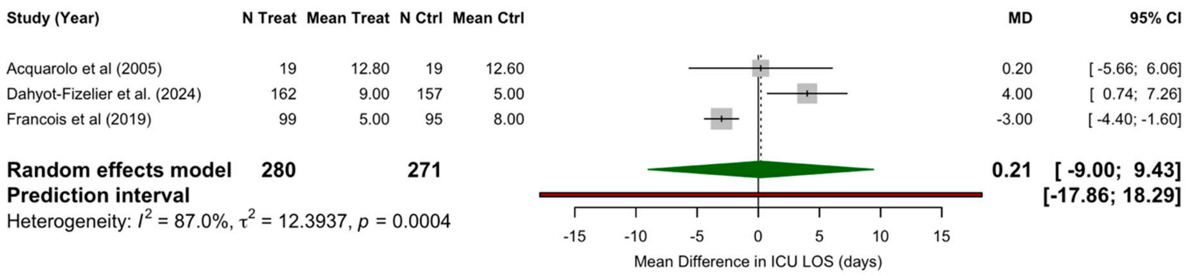

Fig. S7. Forest plot of ICU LOS [3,15,17].

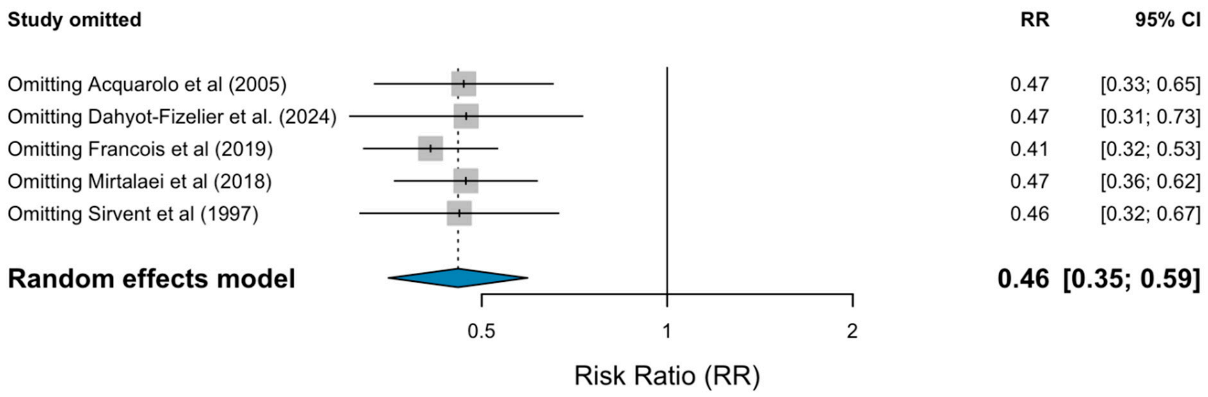

Fig. S8. Leave-one-out sensitivity analysis of EO-VAP incidence [3,7,15-17].

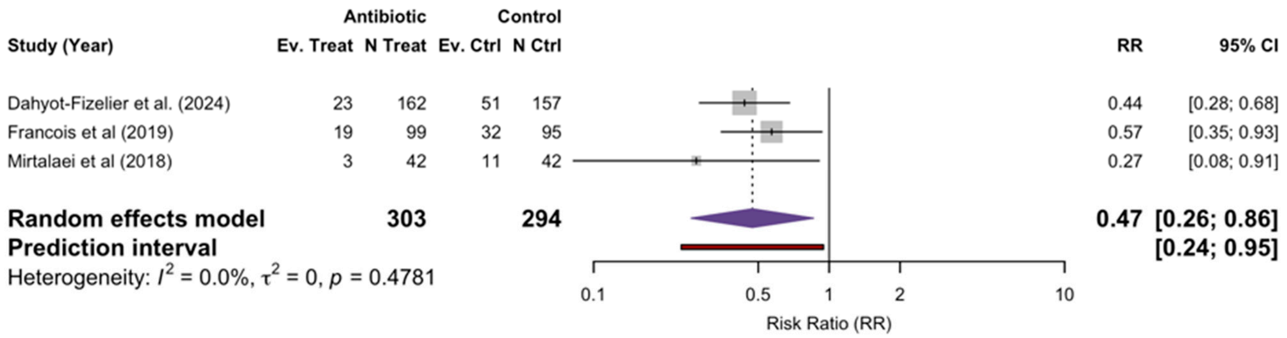

Fig. S9. Forest plot of EO-VAP incidence restricted to the three RCTs published from 2015 onward [3,15,16].

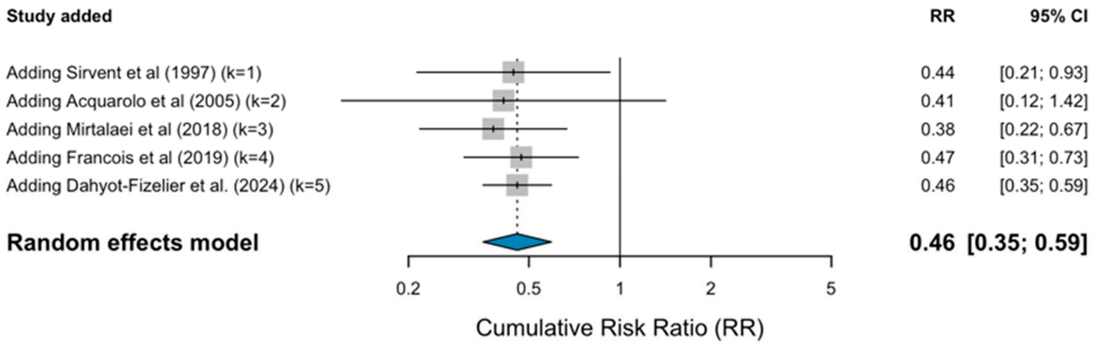

Fig. S10. Forest Plot of cumulative meta-analysis of EO-VAP incidence in chronological order of publication [3,7,15,16,17].

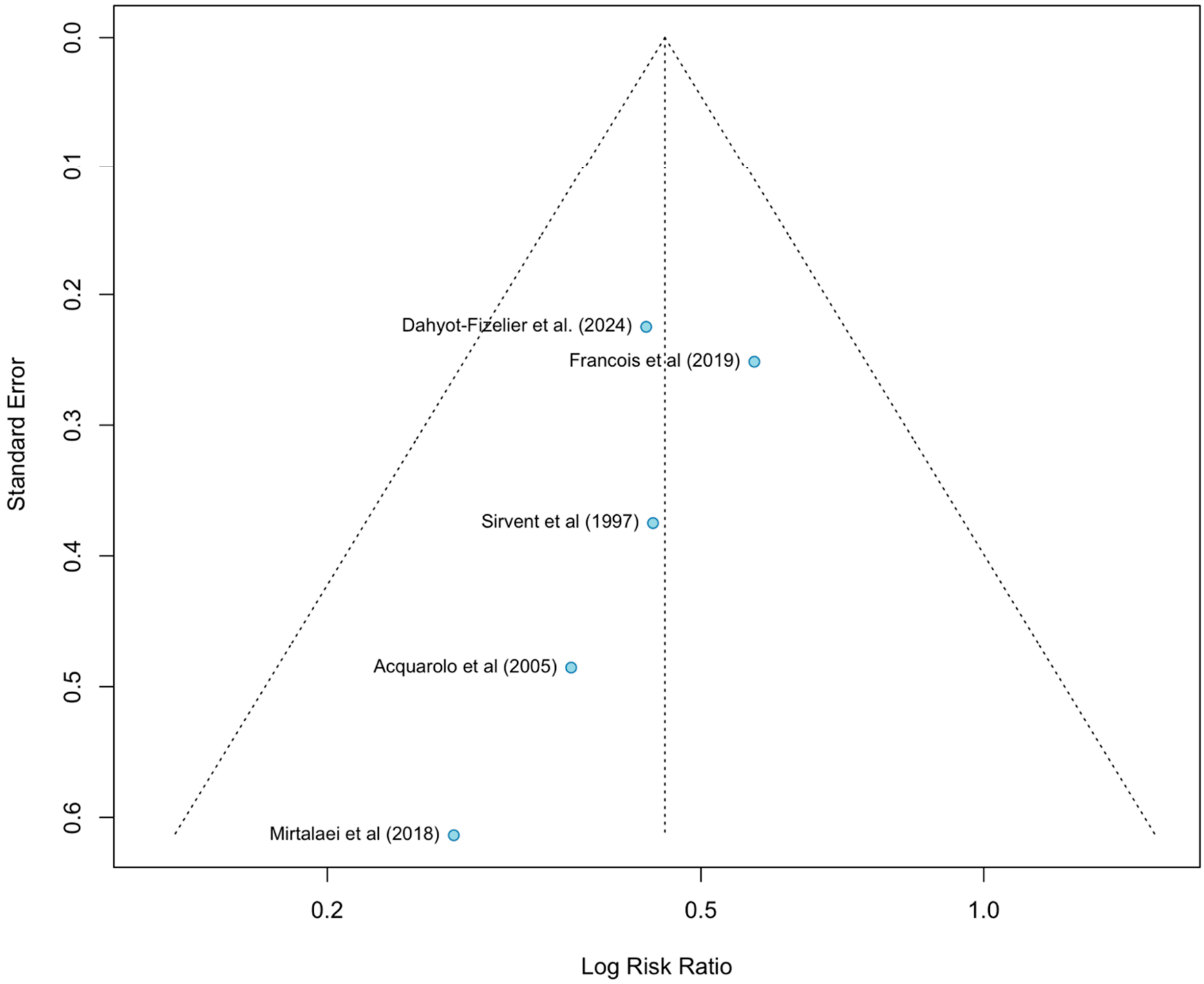

Fig. S11. Funnel plot of the five RCTs included in the primary meta-analysis [3,7,15,16,17].

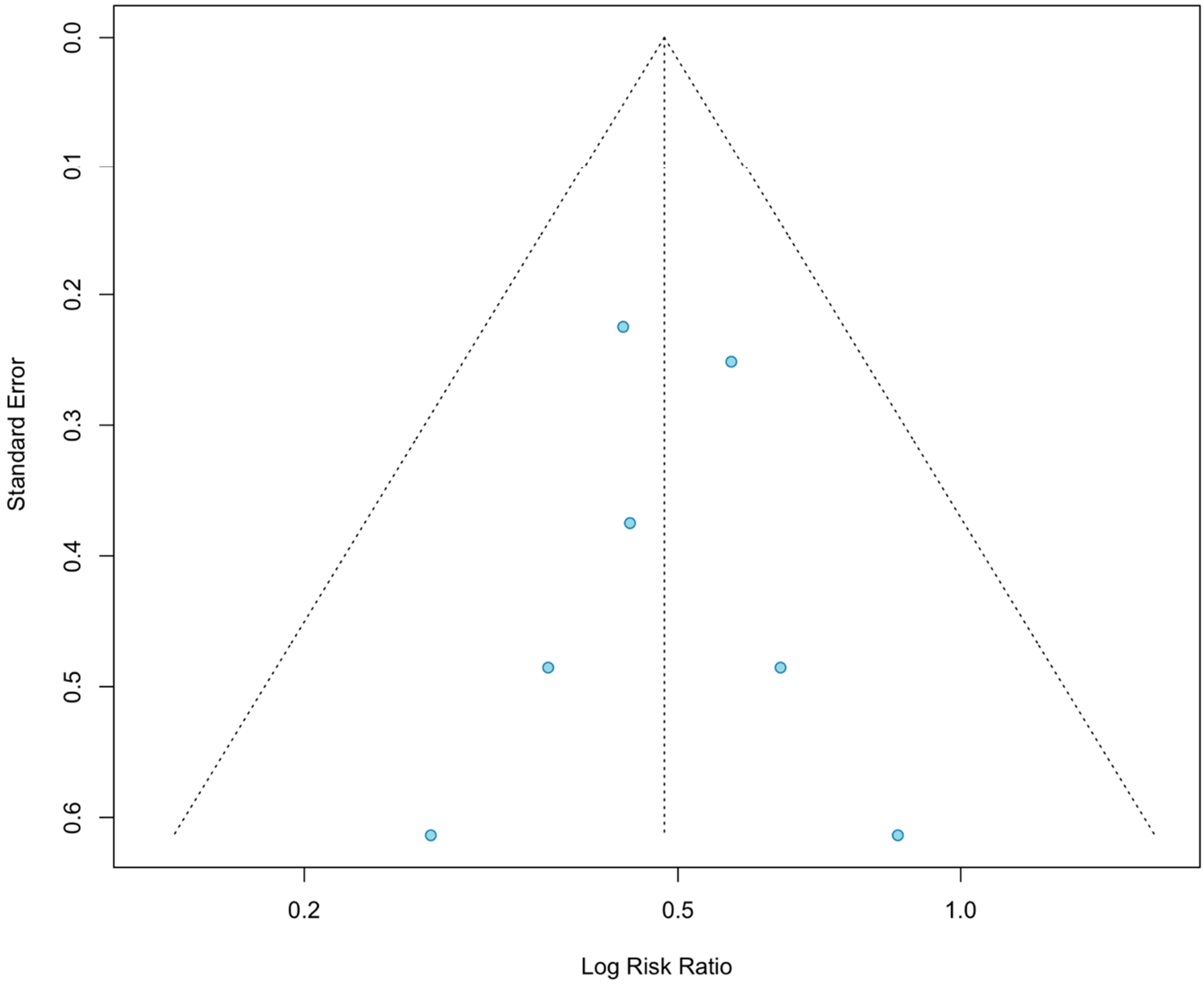

Fig S12. Funnel plot after application of the trim-and-fill method (L-estimator).

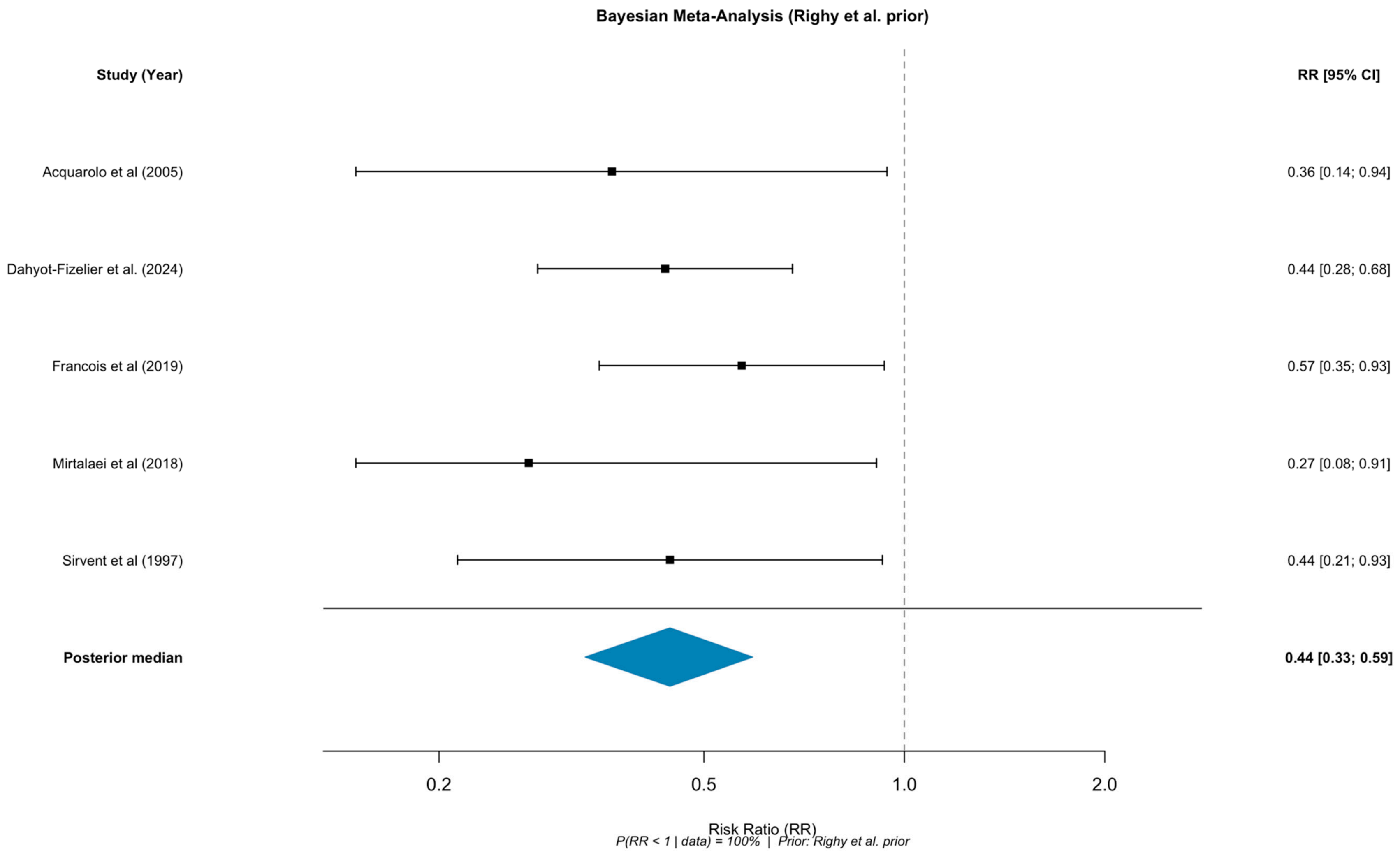

Fig. S13. Posterior density distribution of the effect size (log RR scale) from the Bayesian analysis using the Righy et al. [10] prior [3,7,15-17].

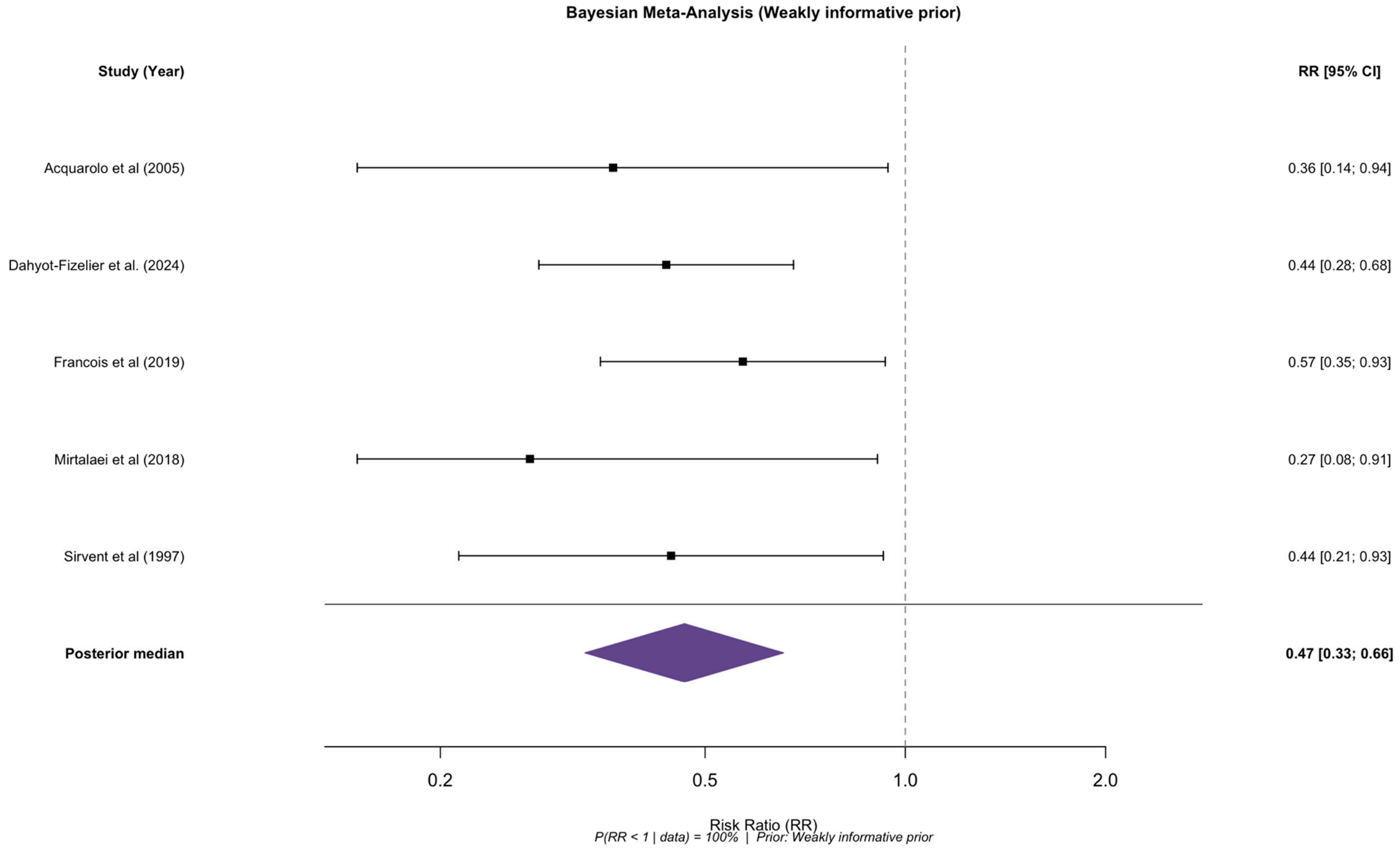

Fig. S14. Posterior density distribution of the effect size (log RR scale) from the Bayesian sensitivity analysis [3,7,15-17].
